# CCA-1.1, a Novel Curcumin Analog, Exerts Cytotoxic anti-Migratory Activity toward TNBC and HER2-Enriched Breast Cancer Cells

**DOI:** 10.31557/APJCP.2021.22.6.1827

**Published:** 2021-06

**Authors:** Dhania Novitasari, Riris Istighfari Jenie, Rohmad Yudi Utomo, Jun-ya Kato, Edy Meiyanto

**Affiliations:** 1 *Cancer Chemoprevention Research Center, Faculty of Pharmacy, Universitas Gadjah Mada, Yogyakarta, Indonesia. *; 2 *Macromolecular Engineering Laboratory, Department of Pharmaceutical Chemistry, Faculty of Pharmacy, Universitas Gadjah Mada, Sekip Utara, Yogyakarta, Indonesia. *; 3 *Medicinal Chemistry Laboratory, Department of Pharmaceutical Chemistry, Faculty of Pharmacy, Universitas Gadjah Mada, Sekip Utara, Yogyakarta, Indonesia. *; 4 *Laboratory of Tumor Cell Biology, Nara Institute of Science and Technology, Ikoma, Nara, Japan. *

**Keywords:** Curcumin analog (CCA-1.1), cell migration, MMP, HER2-enriched breast cancer, TNBC cells

## Abstract

**Objective:**

Chemoprevention curcumin Analog-1.1 (CCA-1.1) demonstrates antineoplastic effect toward cancer cells. By using triple-negative breast cancer (TNBC), 4T1, and human epidermal growth factor receptor 2 (HER2)-enriched metastatic cells (MCF-7/HER2), we evaluate the cytotoxic and antimigration activities from CCA-1.1.

**Methods:**

The cytotoxic activities from a single treatment of CCA-1.1 and in combination with doxorubicin were determined through MTT assay. We also calculated the selectivity index and combination index of CCA-1.1 from the cytotoxic data. Migrating cells were evaluated using wound healing assay, and the MMP2 and MMP9 secretion levels were determined through gelatin zymography.

**Results:**

As hypothesized, CCA-1.1 performed cytotoxic activity during treatment in 4T1 and MCF-7/HER2 cancer cells with good selectivity (Selectivity Index >2). In addition, CCA-1.1 demonstrated a synergistic effect in combinatorial treatment with a low dose of doxorubicin. A single treatment of CCA-1.1 repressed cell migration in 4T1 and MCF-7/HER2 cells. Under gelatin zymography, CCA-1.1 subsided the activities of MMP-9, thereby revealing the potency of CCA-1.1 as an anti-migratory agent. Moreover, MMP-9 was also eminently expressed in TNBC and HER2-enriched breast cancer cells when compared with that in other subtypes.

**Conclusion:**

Our preliminary study collectively reinforces the potential effect of CCA-1.1 through the inhibition of highly aggressive cell migration, particularly in breast cancer.

## Introduction

**Figure 1:**
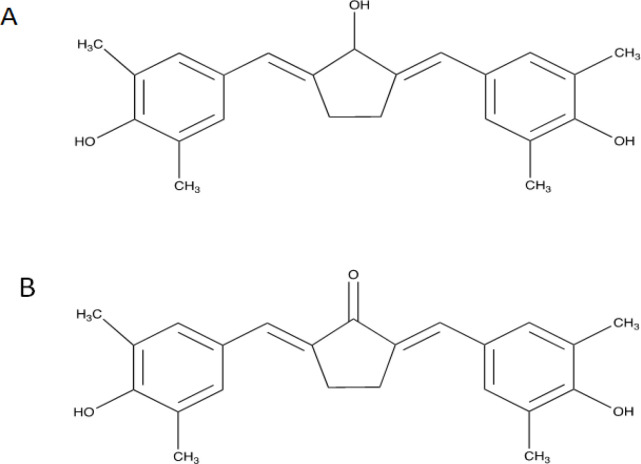
Chemical Structure of A. CCA-1.1 (2,5-bis-(4-hydroxy-3,5dimethylbenzylidene)cyclopentanol) and B. PGV-1 (2,5-bis-(4-hydroxy-3,5-dimethylbenzylidene)cyclopentanone)

In the recent years, considerable numbers of cutting-edge studies have been performed using transcriptomic and proteomic technologies to investigate specific oncological pathways that has escalated the development of new drug targets (Armando et al., 2020). Currently, we developed CCA-1.1 (Figure 1A) that could achieve more stable chemical and physical features compared to its parent compound (PGV-1 and curcumin). CCA-1.1 demonstrated anticancer activities in vitro in an experiment with K562 leukemic cells, TNBC 4T1 cells, T47D, and MCF7 breast cancer cells, as well as colorectal WiDr cancer cells, with high selectivity in non-cancerous cells (Novitasari et al., 2021a, 2021b; Wulandari et al., 2020c, 2021). In TNBC 4T1 and estrogen receptor (ER)+ MCF-7 cells, CCA-1.1 induced mitotic arrest and increased the ROS level to induce cell senescence (Novitasari et al., 2021a). In luminal T47D cells, CCA-1.1 treatment led to cell accumulation in the G2/M phase, which synergistically enhanced the apoptosis effect of doxorubicin (dox), as well as inhibited cell migration (Wulandari et al., 2020b, 2021). CCA-1.1 also generated intracellular ROS, which resulted in cell senescence and cell cycle arrest in colorectal cancer cells (Wulandari et al., 2020c), and the possible target was also documented through a bioinformatics approach (Wulandari et al., 2020a). Moreover, CCA-1.1 performed better than PGV-1 in a molecular docking study, suggesting a higher binding affinity toward several ROS scavengers (Utomo et al., 2021). These reported data demonstrated the impressive results of CCA-1.1 for the further elucidation of its molecular mechanism in different approaches against cancer cells.

Cancer is driven by genetic and epigenetic transformations, and alteration can be spotted regarding dysregulation in broader signaling pathways that promote tumor progressions, such as alteration in the tumor microenvironment that often leads to invasion and metastasis (Sever and Brugge, 2015). In case of breast cancer, for example, up to 70% of the cells presented with positive immunohistochemical detection for ER and or progesterone receptor, while the other subtypes such as human epidermal growth factor receptor 2 (HER2)-enriched comprises of approximately 12%–20% and triple negative breast cancer (TNBC) cases that presented with 15%–20% in invasive breast cancer (Fragomeni et al., 2018). Despite the low percent, HER2-enriched and TNBC subtypes have been associated with worse prognosis due to the heterogeneity in tumors (Fragomeni et al., 2018; Godoy-Ortiz et al., 2019). The overexpression of HER2 promotes aberrant tumor proliferation and fosters an invasive and metastasis phenotype (Freudenberg et al., 2009). The TNBC subtype contributes to a high risk of metastasis considering the tumor cells’ aggressivity and the lack of target for therapy (Rakha and Chan, 2011). One characteristic of cancer that requires attention is the ability to penetrate the blood vessels and invade distant organs, in other words, metastasis (Krakhmal et al., 2015). Numerous data reveal that matrix metalloproteinases (MMPs) play a pivotal part in cancer metastasis due to the enzyme’s capability to diminish the extracellular matrix (ECM) and destroy the tumor microenvironment (TME), which allows cancer cells to migrate (Lv et al., 2018). Mainly, MMP-2 and MMP-9 are most well-studied in cancer cells since these MMPs are highly secreted in breast cancer cells as a proenzyme before activation through hydrolysis and degrade collagen type-IV basement membrane (BM), which in turns alters the ability of BM to disrupt cancer cell mobility (Li et al., 2017; Zhang et al., 2014). This study uses 2 types of breast cancer: 4T1 cells, the TNBC model, and MCF-7 transfected with HER2 gene or MCF-7/HER2 cells, representing HER2-enriched breast cancer cells. These two cancer cells have known for the elevated MMP-2 and MMP-9 expressions (Li et al., 2017), which is relevant to the aim of the present study. There is still no available antineoplastic drug targeted to MMP-9 because the cytotoxic effect was not sufficiently strong to kill cancer cells and have low solubility (Lv et al., 2018).

Our study demonstrated that CCA 1.1 exerts cytotoxic activity toward 4T1 and MCF-7/HER2 cells in single and combination treatments with dox. This study explored the effect of CCA-1.1 treatment on cancer cell migration and the proteolytic activity of MMPs toward highly invasive breast cancer cells. The results of this study expectantly promotes CCA-1.1 as an anticancer agent targeted with TNBC and HER2-enrichment breast cancer subtypes. Therefore, when taken together, the study data along with prior reports support the evidence of CCA-1.1 as a potential chemotherapeutic agent with multiple targets and good selectivity for breast cancer therapy.

## Materials and Methods

*Chemical compounds*

CCA-1.1 and PGV-1 used in this study were procured from Cancer Chemoprevention Research Center, Faculty of Pharmacy, Universitas Gadjah Mada. We used dox hydrochloride (dox) powder from Sigma Aldrich (California, MO, USA). All the tested compounds were diluted in dimethyl sulfoxide (Merck, Germany) before use in each experiment.

*Cell culture*

The 4T1, MCF-7/HER2 breast cancer cells, and 3T3 fibroblast cells were kindly provided by NAIST and used throughout this study. The cells were maintained in high-glucose DMEM (Gibco Life Technologies, CA, USA) supplemented with 10% fetal bovine serum (Gibco Life Technologies) and 1.5% penicillin-streptomycin (Gibco Life Technologies), followed by storage at 37°C with 5% CO_2_ for propagation. The cells were harvested with trypsin-EDTA (Gibco Life Technologies) and split in a new tissue culture dish or seeded in a well plate for use in further experiments.

*Cytotoxic assay*

The single and combination cytotoxic activities were measured by the MTT assay as suggested by past studies with slight modification (Novitasari et al., 2021b; Wulandari et al., 2021). Briefly, the cells were harvested and plated in a well plate, followed by incubation for 24 h before the treatment. Several concentrations ranging from 0.5 to 20 µM (in 4T1 and MCF-7/HER2 cells) and 2–50 µM (for 3T3 cells) of CCA-1.1 and PGV-1 were diluted within the culture medium for 24 and 48 h treatments. The medium was withdrawn and replaced with 0.5 mg/mL MTT (Biobasic) for 4 h; the enzymatic reaction was then terminated with SDS-HCl (0.01 N) and stored overnight; the cells were then observed for their absorbance with a microplate reader (Biorad). The absorbance data (n = 3) were converted to count the IC_50_ value.

The selectivity index (SI), which indicated the cytotoxic selectivity for CCA-1.1, was calculated from the IC_50_ of CCA-1.1 in normal cells as per IC_50_ CCA-1.1 in cancer cells. The SI value >2 was considered to indicate that the compound had high selectivity (Machana et al., 2011; Prayong et al., 2008; Rashidi et al., 2017). For the combinatory treatment assay, we used Chuo-Talalay method (Chou, 2010) and calculated the combination index (CI) value, as previously reported (Ikawati et al., 2020; Yunita et al., 2020).

*Migration assay*

We assessed 4T1 and MCF-7/HER2 cells’ migratory activities under CCA-1.1 treatment by the scratch-wound assay. The 4T1 cells (8.0 × 10^4^/well) and MCF-7/HER2 (8.8 × 10^4^/well) were separately seeded in a 24-well plate. The next day, the cells were starved overnight in a starvation medium (0.5% FBS). A wound was created in the center of the bottom dish of the well using a sterile yellow tip and then washed with PBS to eliminate the unattached cells. We treated the wells with corresponding doses of compounds dissolved in the medium and placed into the wells. The well plate was stored in the incubator, and images were documented at specific time intervals. The gap closure was quantified from the measurement of the wound gap from each observation time and defined as the closure percent by using ImageJ software (version 1.51) (Qodria et al., 2018; Ramadani et al., 2018).

*Gelatin zymography*

We determined the proteolytic activity level of MMPs by gelatin zymography (GZ) and performed using the technical procedure for the assay, as suggested elsewhere with some modification (Ikawati et al., 2020; Jenie et al., 2018; Meiyanto et al., 2021). After treatment with the tested compounds using the starvation medium, the culture medium was collected and then used as the samples for GZ. Briefly, 20 μL of the prepared protein lysates from each treatment group was separated through 0.1% gelatin of SDS-polyacrylamide gel (PAGE). After gel incubation for 24 h, we colored the same with Coomassie blue for 30 min, followed by destaining with methanol-acetic acid solution for 1  h. Clear bands on the dark gel indicated gelatin degradation. The analysis of gelatinase activity was quantified by using the ImageJ version 1.51.

*Data mining of MMP-9 and MMP-2*

The mRNA expression levels of MMP-9 and MMP-2 in breast cancer and normal cells were generated using Oncomine (http://www.oncomine.org), with the following screening conditions as followed: i) Cancer type: Breast; ii) gene: MMP-9 or MMP-2; iii) data type: mRNA; iv) analysis type: cancer vs. normal analysis; v) clinical outcome: survival status; and vi) threshold setting conditions by default (Rhodes et al., 2004).

We also used the Breast Cancer Gene-Expression Miner (bc-GenExMiner; version 4.5) (updated in June 2020) (http://bcgenex.ico.unicancer.fr) to assess the expression of MMPs in comparison with the clinical parameters, including TNBC, HER2, p53 status, and the Sorlie’s subtypes. According to different clinicopathological parameters, Welch’s test was automatically acquired in the program to compare the abnormal expression of MMPs between patient groups as stated earlier. p < 0.05 from statistical analysis indicated significant difference (Jézéquel et al., 2012).

*Statistical analysis*

The data reported in the study were demonstrated as the mean of 3 data ± standard error (SE). We assessed one-way analysis with variance (ANOVA) (p < 0.05) continued by post-hoc Dunnett’s test to define the differences between the groups by using the RStudio version 1.3 (RStudio, PBC).

## Results

*CCA-1.1 suppressed the proliferation of 4T1 and MCF-7/HER2 cancer cells*

**Figure 2:**
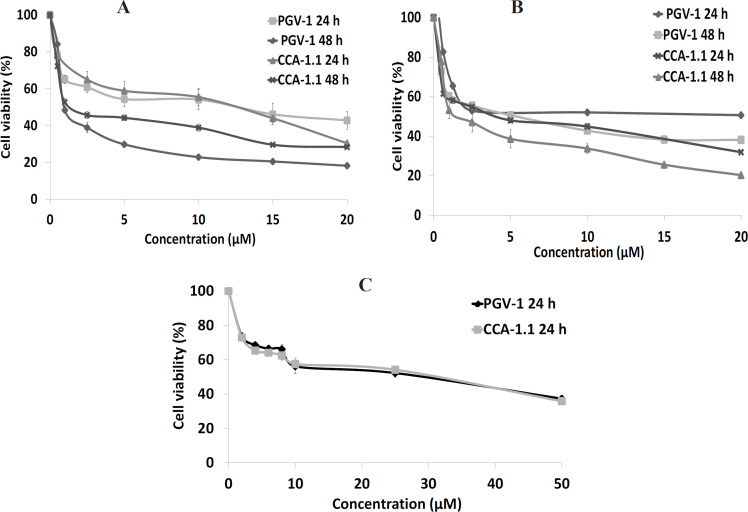
Cytotoxic Effects of CCA-1.1 and PGV-1 on 4T1 Cells (A), MCF-7/HER2 Cells (B), and 3T3 Cells (C). Cells were seeded in a well-plate and incubated for 24 h before treatment with either CCA-1.1 or PGV-1. The cell viability was evaluated using MTT assay. The figure shows the graph plotted between concentration and cell viability of CCA-1.1 and PGV-1 in 4T1 cells, MCF-7/HER2 cells, and 3T3 cells. The data is presented as the mean ± SE from 3 replications

Our study aimed to scrutinize the anticancer effects from CCA-1.1 against TNBC 4T1 cells and HER2-positive using MCF-7/HER2 breast cancer cells. Hence, we assessed the single treatment of CCA-1.1 (with PGV-1 as reference) against 4T1 and MCF-7/HER2 cells (Figure 2A and 2B) that demonstrated the cytotoxic activity with IC_50_ values after 24 and 48 h of treatment (Table 1). The IC_50 _of CCA-1.1 after 24 h of treatment were 10 and 6 μM on 4T1 and MCF-7/HER2 cells, respectively. Next, we prolonged the CCA-1.1 to 48 h of treatment, which subsequently resulted in the IC_50_ values of 1 and 4 μM on 4T1 and MCF-7/HER2 cells, respectively. The cytotoxic effect of CCA-1.1 was similar to that of PGV-1 when treated in 4T1 cells, while it seemed different when treated in MCF7/HER2 cells. In a previous study, we reported the IC_50_ for CCA-1.1 in 4T1 cells (3 μM), which was almost 1/3-fold of the current data, which may be because of the use of a different method for estimating cell viability activity, for instance, a previous study used trypan blue exclusion assay, while the present one used the MTT assay. The use of different approaches based on the 2 methods (trypan blue test evaluated cell viability through an imbalance of cell membrane and MTT assay determined viable cells through mitochondria activities) may have given rise to different IC50 values CCA-1.1.

In chemotherapy development, the selectivity becomes a critical feature, indicating that a drug candidate should exhibit higher effect against cancer cells than normal cells, with SI >2 marked as high selectivity of a candidate drug (Machana et al., 2011; Rashidi et al., 2017). Therefore, we also assayed the viability assay from CCA-1.1 on 3T3 cells (immortalized fibroblast cells) and quantified their SI. As shown in Table 2, we calculated CCA-1.1 (also PGV-1) with SI value >2 in 4T1 and MCF-7/HER2 cells, suggesting that the compound has good selectivity and can be considered for further experiments to elucidate its molecular mechanisms.

*CCA-1.1 enhanced the cytotoxicity effect from Dox on breast cancer cells*

**Figure 3:**
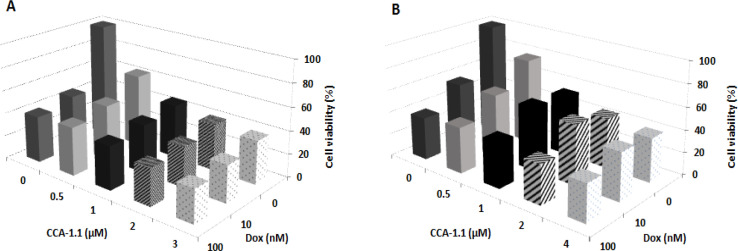
Combinational Cytotoxic Activity of CCA-1.1 against 4T1 Cells (A) and MCF-7/HER2 Cells (B). Cells were seeded in a 96-well plate, incubated for 24 h, and then treated with a combination of doxorubicin (Dox) with CCA-1.1. The cell viability was determined using MTT assay. The graph was plotted using the mean values from 3 replications

In this study, we used CCA-1.1 in combination with dox, which is the standard golden chemotherapy for breast cancer. Nevertheless, the challenges of the resistance occurred in the clinical cases. A combinatorial treatment was designed by adding CCA-1.1 and dox together on 4T1 and MCF-7/HER2 cells so as to enhance the effectiveness for dox. We assessed the CI value to determine the effect from combination treatment, with CI value <0.9 presented as a synergistic effect from the treatment. The CCA-1.1 enhanced the cytotoxicity from dox (Figures 3A and 3B) in both 4T1 and MCF-7/HER2 cells, and the combination treatment also displayed similar viable cells percent in both the cell types. Moreover, we confirmed, based on the CI value, that the combination at all tested concentrations were synergistic in both 4T1 and MCF-7/HER2 cells (Tables 3 and 4). These findings suggested that CCA-1.1 can be paired with dox for combinatorial chemotherapy against breast cancer cells.

*CCA-1.1 inhibited cells migration of 4T1 and MCF-7/HER2 cancer cells*

**Figure 4:**
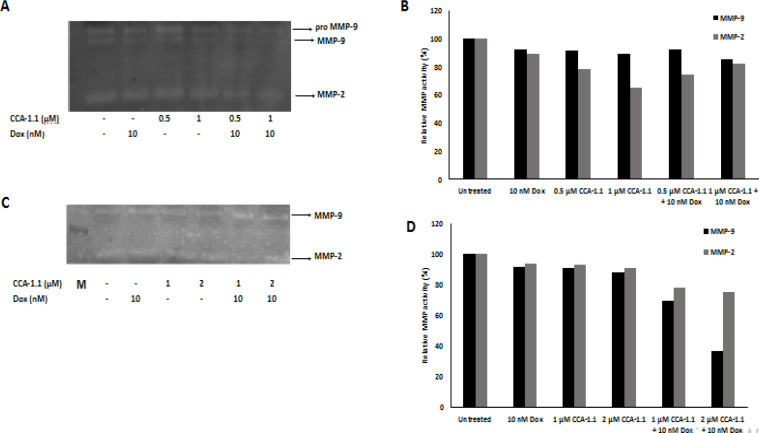
The Effect of CCA-1.1 on Cell Migration. The 4T1 and MCF-7/HER2 cells were scratched then treated with CCA-1.1 at indicated concentration alone and in combination with Doxorubicin (Dox). The morphology of the 4T1 cells (A) and MCF-7/HER2 cells (B) after scratched and treated with the compounds under an inverted microscope with magnification of 100x. The percentage of 4T1 cells (C) and MCF-7/HER2 cells (D) closure after treatment. The area of closure was measured using ImageJ (n=3) and % closure was calculated. The asterisk (*) indicate statistical difference compared to % closure of control (untreated cells) at the same hour of observation (p<0.05)

We determined the CCA-1.1 effect after treatment on cell migration using metastatic breast cancer cell models. We used low concentration of dox to induce cells migration (Amalina et al., 2017; Meiyanto et al., 2021); the effect can be seen in Figure 4. Meanwhile, CCA-1.1 demonstrated the inhibitory effect at a dose of 0.5 μM in 4T1 cells and 1 μM in MCF-7/HER2 cells (Figure 4). The combination of CCA-1.1 with dox resulted in a lower percent of closure when compared to that with dox alone. These results indicated that CCA-1.1 potentially inhibited the cell migration rather than dox, and CCA-1.1 performed a superior effect on both 4T1 and MCF-7/HER2 cells.

*CCA-1.1 decreased the secretion level of MMP-9 and MMP-2 on breast cancer cells*

**Figure 5:**
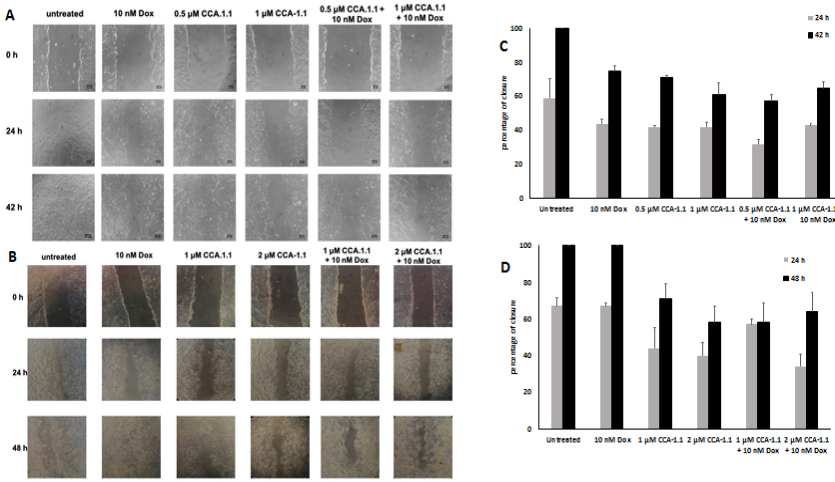
The Effect of CCA-1.1on the secretion of MMP-2 and MMP-9. The 4T1 cells (A) and MCF-7/HER2 cells (C) were treated with CCA-1.1 in alone and combination with Doxorubicin at the indicated concentration for 24 h. The band profiles of MMP-2 and MMP-9 expression in (B) 4T1 cells and (D) MCF-7/HER2 cells. The band intensity was quantified using Image J

After we realized that CCA-1.1 could inhibit cell migration against these cancer cells, we wondered if the level of MMPs were also affected upon the treatment with CCA-1.1. We know that cancer cell migration and invasion to neighboring tissues are a critical part of metastasis cascade. These processes involve the role of MMP-9 and MMP-2 by altering the ECM components, which causes the cancer cells to invade the neighboring cells (Gialeli et al., 2011; Kessenbrock et al., 2010; van Zijl et al., 2011). Therefore, we evaluated the secretion level of MMP-2 and MMP-9 after treatment with CCA-1.1. We also realized that treatment with CCA-1.1 suppressed the activity from respected MMPs in both 4T1 and MCF-7/HER2 cells (Figure 5). In 4T1 cells, CCA-1.1 treatment reduced up to 35% of MMP-9 activity against the untreated group. We therefore suggest that CCA-1.1 can be used to suppress cancer cell migration, which is mediated by the repression of the MMP-9 activity.

*Data mining analysis of MMP-9 and MMP-2 in breast cancer*

**Figure 6:**
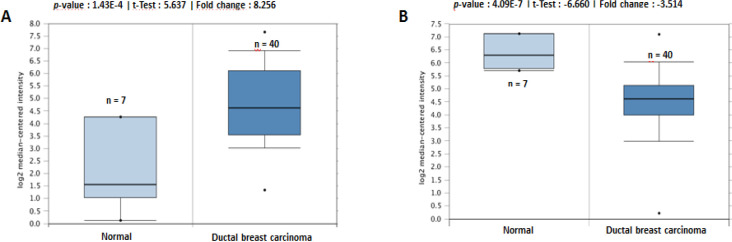
Box Plots Retrieved from Gene Expression Data in Oncomine Comparing mRNA Expression in Normal and Melanoma Tissue. The p-value was set at 0.01 and fold change was defined as 2. (A) Comparison of MMP-9 expression in ductal breast carcinoma (n=40) and normal tissue (n=7) (B) Comparison of MMP-2 expression in ductal breast carcinoma (n=40) and normal tissue (n=7).

**Figure 7:**
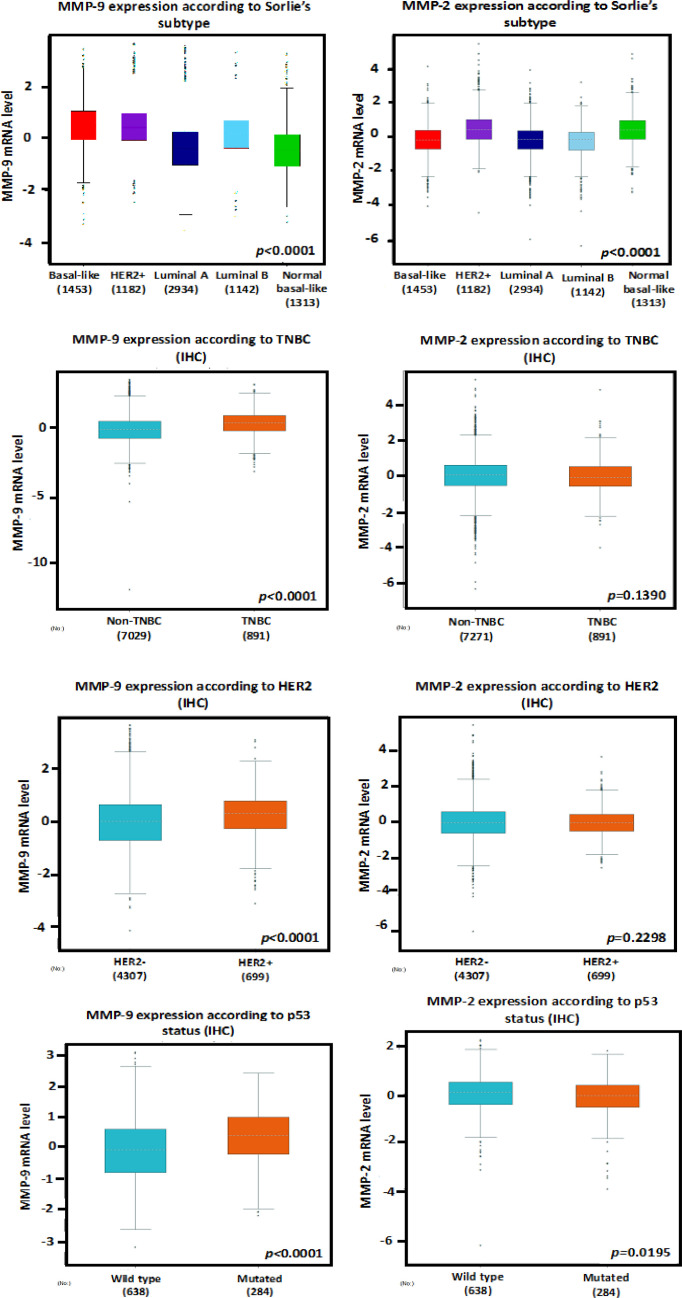
Association between the mRNA Expression Levels of MMP2/9 with Sorlie’s Subtype, TNBC, HER2, and p53 status of breast carcinoma. The data of IHC samples from bc GenExMiner datasets were analyzed. p < 0.05 was considered to be statistically significant

Through Oncomine analysis, we revealed that, in a dataset from Richardson et al. (Richardson et al., 2006), MMP-9 was highly expressed in several types of breast cancer relative to that in normal tissues. The fold change was 8.256 in ductal breast carcinoma (Figure 6A), and the other datasets also demonstrated a similar profile (Xia et al., 2019). On the other hand, the expression of MMP-2 tended to be lower in breast carcinoma against that in normal tissues, with a fold change of -3.514 (Figure 6B), as suggested by in vitro studies, while MMP-2 and MMP-9 were highly expressed in breast cancer tissues and associated with worsening prognosis (Li et al., 2017). Therefore, we attempted to determine the transcriptional levels of MMP-9 and MMP-2 by using the Bc-GenExMiner software using the TCGA dataset. The expressions of MMP-9 and MMP-2 were higher in basal-like (similar to TNBC) and HER2+ compared with those in other subtypes such as luminal A or B (Figure 7).

Moreover, MMP-9 was also positively associated with the HER2 and TNBC status. In terms of the p53 status, the MMP-9 level was higher in mutated cells than in the wild types. This study also denoted a significant rise in the MMP-9 expression in patients with TNBC and HER-2 positivity. These bioinformatics analyses identified MMP-9 as a potential marker to be developed as a specific target for breast cancer therapy.

**Table 1:** The IC_50_ Value of Curcumin Analog PGV-1 and CCA-1.1 against 4T1, MCF-7/HER2, and 3T3 Cells

Compound	IC_50_ value (µM)				
	4T1 cells		MCF7/HER2 cells		3T3 cells
	24 h	48 h	24 h	48 h	24 h
PGV-1	12	2	16	9	45
CCA-1.1	10	1	6	4	43

**Table 2:** Selectivity Index (SI) of PGV-1 and CCA-1.1

Cells	Selectivity Index (SI)	
	PGV-1	CCA-1.1
4T1	3.8	4.3
MCF7/HER2	2.8	7.2

**Table 3:** Combinatory Index (CI) Value of Combinational Cytotoxic Assay from CCA-1.1 and Doxorubicin in 4T1 Cells

CCA-1.1 (µM)	Doxorubicin (nM)	
	10	100
0.5	0.21	0.41
1	0.2	0.41
2	0.25	0.42
3	0.32	0.47

**Table 4:** Combinatory Index (CI) Value of Combinational Cytotoxic Assay from CCA-1.1 and Doxorubicin in MCF-7/HER2 Cells

CCA-1.1 (µM)	Doxorubicin (nM)	
	10	100
0.5	0.17	0.36
1	0.24	0.39
2	0.33	0.4
4	0.39	0.52

## Discussion

The present study aimed to determine a novel candidate as an anticancer agent with multiple targets in the cancer pathway that can overcome the limitations during therapy (Wang et al., 2019). We previously revealed that a new curcumin analog, CCA-1.1, promotes anticancer potential in several breast cancer cells and colorectal cancer through cell cycle arrest and ROS generation (Novitasari et al., 2021b, 2021a; Wulandari et al., 2021). Currently, we reported the preliminary studies of anti-migratory activity from CCA-1.1 against TNBC and HER2+ breast cancer cells. We used 2 different types of breast cancer cells to evaluate the antimigration activities of CCA-1.1, as these subtypes promote aggressive tumor growth, lead to malignant cell migration and invasion, and mediate metastasis, resulting in poor prognosis; all of these with their distinct mechanisms and pathways (Rakha and Chan, 2011).

Here, our data demonstrated that the cytotoxicity of CCA-1 was equivalent, even somewhat more than that of PGV-1 in 4T1 and MCF-7/HER2 cancer cells, which is represented as the TNBC and HER2 positive (HER2+) model, respectively. These data correlate with previous findings supporting this claim. Moreover, CCA-1.1 also demonstrated a synergistic effect with dox, which has raised hopes for CCA-1.1 potency for use in either single treatments or in combination with the existing chemotherapeutic agent dox in the future.

Next, we investigated whether CCA-1.1 also promotes the inhibition of cancer cells migration and its involvement in MMPs activities against TNBC and HER2+ breast cancer cells, considering that numerous studies have revealed that MMPs (specifically MMP-9 and MMP-2) are upregulated on these breast cancer cell models and secreted to the ECM, which in turn permits cancer cells to invade the neighboring tissues (Li et al., 2017; Lv et al., 2018; Nanda et al., 2013; Zhou et al., 2014). CCA-1.1, at the dose of 0.5 μM, attenuated cell migration and reduced the MMP-9 activity (Figures 4A and 5A) in 4T1 cells. These phenomena also occurred in MCF-7/HER2 cells (Figure 4B and 5B) at a concentration of 1 μM. These data together provide more evidence of CCA-1.1’s activity in inhibiting cell migration against ER+ T47D cells (Wulandari et al., 2021). Following these findings, PGV-1 also provided with a similar anti-migratory effect against both 4T1 and MCF-7/HER2 cells (Meiyanto et al., 2021, 2019), assuming that the structural change did not affect its anticancer properties. Although this information is limited, the authors provided fascinating insights about its potency as an anti-metastatic agent for application in future development. We realize that our collected data are not coherently designed to demonstrate the comprehensive relationship between each data. Therefore, further studies are warranted to explore the underlying molecular mechanisms.

In more detail, our findings demonstrated that CCA-1.1 tended to inhibit stress-induced dox against these metastatic tumor cells. Moreover, Amalina et al., (2017) denoted that dox formed lamellipodia in these 2 breast cancer types, and, consistently, the effect of dox in promoting migration was revealed by Liu et al., (2019) in MCF-7 and BT474 cells. This information promotes the merit points for CCA-1.1 to be developed as novel anti-metastasis agents in the future, in which CCA-1.1 also intended to be less toxic in immortalized fibroblast cells, as also demonstrated in this study.

Increasing reports have established the relationship between MMPs activity and breast cancer, which has become an exciting topic. Therefore, finding an anticancer agent that targets MMPs can be a reference indicator for metastatic breast cancer therapy (Li et al., 2017). Prior studies have revealed the CCA-1.1 parent compound PGV-1 exerted an anti-migratory effect and lowered the MMP-9 and Rac-1 expressions in TNBC 4T1cells; this phenomenon was also presented in HER2+ breast cancer cells (Meiyanto et al., 2021, 2019). Here, we reported that CCA-1.1 also exhibited a similar pattern in inhibiting cancer cell migration and in reducing MMPs activities against 4T1 and MCF-7/HER2 breast cancer cells. Thus, we offered that the MMP-9 in cancer cells should be constructed as a novel target for an anticancer drug to treat malignant phenotype tumors, which are mainly characterized by HER2-positive and TNBC subtypes (Mehner et al., 2014; Yousef et al., 2014).

We generated web-based platforms from Oncomine to bc-GenExMiner in order to validate the significance of MMP levels in breast cancer patients. According to the Oncomine database, the MMP-9 expression was significantly elevated in breast cancer. It was then revealed that the MMP-9 expression increased, particularly in HER-2 positive and TNBC patients through the bc-GenExMiner analysis. In addition, the expression of MMP-9 was also elevated in p53-mutation breast cancer cells, which is extremely interesting considering that most aggressive breast cancer cells feature p53 mutation (Zhu et al., 2009). The downregulation of MMP-9 is also controlled by p53, mediated through the blocking of nuclear factor-κB (NF-κB) activity (Liu et al., 2006). Notably, a high level of MMP-9 does not correlate with immense activities from MMP-9 since the inactive proenzyme are released into the matrix and not the active form. In addition, the active MMP-9 can be effectively blocked by tissue inhibitors of metalloproteinase-1 or TIMP-1 (Egeblad and Werb, 2002; Vandooren et al., 2017). This information at least provides an insight about the significance of MMP-9 in TNBC and HER2-enriched human breast cancer.

The current breakthroughs in the development of selective MMP inhibitors also provide a particular mechanism for inhibiting MMPs implicated in cancer metastasis. Therefore, we propose that CCA1.1 is a novel candidate to be developed as an anticancer agent, which acts through its cytotoxic and anti-migratory activities. We presume that these experimental findings should encourage us toward a new challenge to develop CCA-1.1 as an anticancer agent, specifically for metastatic breast cancer treatment.

Our study concluded that CCA-1.1 can effectively inhibit cancer cell proliferation. We also performed a synergistic effect study with dox. Moreover, CCA-1.1 was found to inhibit cancer cell migration mediated through the inhibition of proteolytic activity from MMP-9 in 4T1 and MCF-7/HER2 cancer cells. Thus, CCA-1.1 can be used as a potentially useful anti-metastatic agent in breast cancers.

## Author Contribution Statement

EM devised the project, the main conceptual ideas and proof outline. DN performed most of the experimental studies. RYU prepared the sample for the study. RIJ and JK helped to supervise the finding of the project. DN wrote the manuscript with input from all authors. All authors reviewed the results and approved the final version of the manuscript.
